# Invariant Natural Killer T Cell Therapy Attenuates Systemic Inflammation and Improves Transarterial Chemoembolization Outcomes in Hepatocellular Carcinoma

**DOI:** 10.32604/or.2026.082815

**Published:** 2026-08-13

**Authors:** Xiaoxia Wang, Shuo Wang, Chendi Liang, Huili Wu, Songtao Liu, Jinhuan Wang, Jun Lu

**Affiliations:** 1 Department of Medical Oncology, Beijing You An Hospital, Capital Medical University, Beijing, China; 2 Laboratory for Clinical Medicine, Capital Medical University, Beijing, China

**Keywords:** Invariant natural killer T, inflammation, hepatocellular carcinoma, transarterial chemoembolization

## Abstract

**Background:** Invariant natural killer T (iNKT) cells show promise as immunotherapeutic agents for solid tumors, and our prior study demonstrated that combining iNKT-cell therapy with transarterial chemoembolization (TACE) achieved a 58.3% objective response rate (ORR) in hepatocellular carcinoma (HCC). This study further examines iNKT-mediated immune modulation of post-TACE survival dynamics and associated prognostic biomarkers. **Methods:** Clinical data and peripheral blood samples were obtained from 77 HCC patients in Beijing you’an Hospital between 2018–2023, including 38 receiving TACE alone and 39 receiving combined iNKT-cell/TACE therapy. Serial measurements included: Hematological parameters; Liver function tests [alanine aminotransferase (ALT), aspartate aminotransferase (AST), bilirubin]; Inflammatory markers [C-reactive protein (CRP), neutrophil-to-lymphocyte ratio (NLR)]; Cytokine profiling [Interferon-gamma (IFN-γ), Interleukin-6 (IL-6), Interleukin-10 (IL-10)]. Potential Prognostic factors for progression-free survival (PFS) were identified through univariate/multivariate Cox regression. A risk-score model was derived using significant covariates from the multivariate analysis. Model performance was evaluated by time-dependent receiver operating characteristic (ROC) analysis [Area Under the Curve (AUC) calculation] and Kaplan-Meier survival stratification with log-rank testing. **Results:** The TACE group exhibited decreased lymphocytes but increased neutrophils/monocytes post-treatment, whereas iNKT + TACE maintained stable counts. Systemic inflammation indices [NLR/systemic immune-inflammation Index (SII)/systemic inflammation response index (SIRI)] rose significantly in TACE alone (all *p* < 0.05) but remained stable in iNKT + TACE. Cytokine profiling revealed reduced IL-6/IL-10 (*p* < 0.001/*p* = 0.012) and elevated IFN-γ/tumor necrosis factor-alpha (TNF-α) (*p* < 0.01/*p* < 0.001) in iNKT + TACE vs. TACE. Multivariate analysis identified lymphocyte count (HR = 0.18, 95%CI 0.04–0.76, *p* = 0.02), IL-6 (HR = 2.57, 95%CI 1.03–6.86, *p* = 0.04), and IFN-γ (HR = 0.14, 95%CI 0.02–0.98, *p* = 0.04) as independent PFS predictors. The risk model demonstrated strong discrimination (AUC = 0.891, 95%CI 0.73–1.00), with median PFS of 9.5 vs. 3.0 months for low- vs. high-risk groups (log-rank *p* < 0.01) in iNKT + TACE group. **Conclusions:** iNKT cell therapy stabilizes peripheral lymphocyte counts and attenuates TACE-induced inflammation in HCC. The combined evaluation of lymphocyte levels, IL-6, and IFN-γ represents a promising prognostic biomarker panel for PFS in iNKT + TACE-treated patients.

## 1 Introduction

Hepatocellular carcinoma (HCC) is one of the most common solid malignancies, conveying a 5-year overall survival rate of approximately 10% [1], and it is one of the leading causes of cancer-related mortality worldwide [2]. The primary treatment modalities for HCC include surgery, transcatheter arterial chemoembolization (TACE), and systemic therapy. Among these, TACE is one of the first-line therapies for HCC patients classified as Barcelona Clinic Liver Cancer (BCLC) stage B or C [3]. However, patient responses to TACE therapy exhibit significant clinical heterogeneity [4]. Although TACE induces localized tumor ischemia and necrosis, it also activates regional hepatic inflammatory responses, which may lead to adverse effects such as posttreatment fever and liver function impairment, ultimately impacting patient survival outcomes [5,6]. Therefore, novel combination therapies are crucial for achieving synergistic antitumor effects while maximizing patient quality of life and improving clinical outcomes.

Invariant natural killer T (iNKT) cells are a specialized subset of T lymphocytes characterized by their expression of an invariant T cell receptor (TCR) chain. These cells recognize glycolipid antigens presented by CD1d molecules, thereby initiating downstream immune responses. Activated iNKT cells exert potent antitumor effects through multiple mechanisms, including secretion of interferon-gamma (IFN-γ) and interleukin-4 (IL-4) to mediate direct tumor cell lysis via the perforin or granzyme pathway, as well as via production of TNF-α to trigger tumor apoptosis through both Fas ligand (FasL)- and TNF-related apoptosis-inducing ligand (TRAIL)-dependent pathways, demonstrating significant therapeutic potential for cancer treatment [7]. Moreover, iNKT cells also play a regulatory role in inflammatory responses. Previous studies have shown that iNKT cells protect the liver from carbon tetrachloride (CCL4)-induced acute hepatitis by suppressing neutrophil infiltration [8].

Our previous study [9] compared the antitumor efficacy of iNKT cell therapy in combination with TACE and of TACE monotherapy in HCC patients. Combining iNKT cell therapy with TACE significantly improved the progression-free survival (PFS), objective response rate (ORR), and disease control rate (DCR) in HCC patients. Moreover, patients receiving combination iNKT cell therapy with TACE (iNKT + TACE) have a significantly reduced incidence of treatment-related adverse events (including fever and elevated ALT/AST levels), and they have higher quality of life (QoL) scores, indicating that iNKT cell infusion contributes to better patient well-being than the control treatment. The improved QoL in iNKT-treated patients enhances their tolerance to subsequent therapies, ultimately prolonging overall survival (OS). Notably, iNKT cells possess dual immunomodulatory functions. In addition to exerting direct antitumor effects, iNKT cells also participate in regulating systemic inflammatory responses through their characteristic cytokine secretion profile [10]. Therefore, we hypothesized that adoptively transferred iNKT cells may not only mediate antitumor effects but also ameliorate TACE-induced inflammatory responses. Therefore, the present study compared the changes in clinical indicators, circulating inflammatory indices and cytokines before and after treatment in patients treated with iNKT cells combined with TACE and those treated with TACE alone. This retrospective study sought to clarify the means by which iNKT cell therapy modulates post-TACE inflammatory responses and efficacy in HCC patients.

## 2 Methods

### 2.1 Study Population

We retrospectively reviewed data from HCC patients who underwent iNKT cell treatment and TACE at Beijing You’an Hospital between 2018 and 2023. The inclusion criteria were as follows: (1) aged between 18 and 80 years; (2) confirmed diagnosis of HCC on the basis of imaging or pathological evidence; (3) received TACE therapy or combined iNKT cell and TACE therapy; (4) patients with Child-Pugh A or B liver function and an Eastern Cooperative Oncology Group Performance Status (ECOG) performance status score of 0 or 1; (5) Patients are considered eligible if they have either never received any prior treatment, or have developed recurrent disease after prior chemotherapy or locoregional therapies (e.g., surgery, radiotherapy, transarterial embolization, chemoembolization, radiofrequency ablation, percutaneous ethanol injection, or cryoablation), as long as the last session of such therapy occurred at least 4 weeks prior to study entry. The exclusion criteria were as follows: (1) coexisting HIV, severe infections, autoimmune diseases, or other types of malignancies; (2) history of liver transplantation or other organ transplantation; (3) previous immunosuppressive therapy or history of drug allergies; and (4) severe hypertension or cardiovascular diseases. Patients who met the inclusion and exclusion criteria were enrolled in the study. The iNKT + TACE group consisted of patients who received iNKT cells combined with TACE therapy, whereas the TACE group included patients who underwent TACE therapy alone. The study protocol were approved by the ethics committee of Beijing Youan Hospital (Jing You Ke Lun [2025] No. 134). Due to the retrospective nature of the study, the requirement for informed consent was waived by the committee.

### 2.2 Actual Therapeutic Intervention

**Figure 1:**
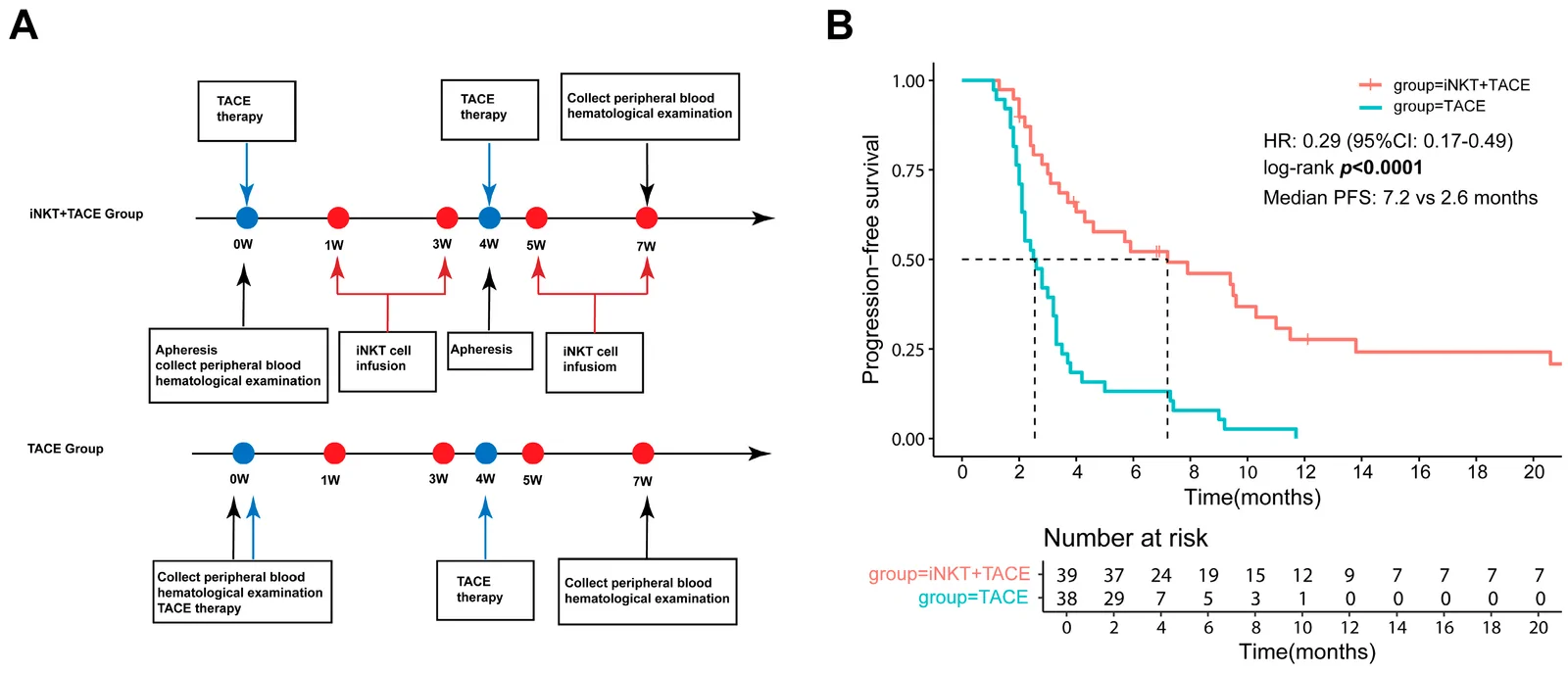
Treatment process and outcomes in the iNKT + TACE group versus the TACE group. (**A**) Flowchart of the treatment and data collection process. (**B**) Kaplan–Meier curves of PFS in patients in the iNKT + TACE and TACE groups. HR: Hazard Ratio.

For the overall treatment procedure, please refer to Fig. 1A. Patients in the TACE group received two TACE treatments at weeks 0 and 4, respectively. The TACE treatment regimen was performed using gelatin sponge, lipiodol and 20 mg of epirubicin. In the iNKT + TACE group, in addition to two TACE treatments, patients underwent two session of iNKT cell therapy after each TACE treatment. All procedures used for the preparation of iNKT cells were detailed in our previous study [11]. For each patient, approximately 6–9 × 10^7^ cells/m^2^ of iNKT cells were intravenously infused, with purity and viability both exceeding 95%.

### 2.3 Collection of Clinical Data

We collected baseline and posttreatment clinical data (at 3 week post-TACE) from the electronic medical record system, including demographic information (such as sex and age), biochemical test data (including liver function Child-pugh, alpha-fetoprotein levels, etc.), BCLC stage, routine blood tests (including lymphocyte counts, neutrophil counts, monocyte counts and platelet counts), and circulating inflammatory indices (neutrophil-to-lymphocyte ratio [NLR], lymphocyte-to-monocyte ratio [LMR], systemic immune-inflammation index [SII], and systemic inflammation response index [SIRI]). The NLR refers to the ratio of neutrophils to lymphocytes, and the LMR refers to the ratio of lymphocytes to monocytes in the peripheral blood. The SII is calculated as follows: SII = platelet count × neutrophil count/lymphocyte count. The SIRI is calculated as follows: SIRI = neutrophil count × monocyte count/lymphocyte count. The time from the date of treatment initiation to tumor progression was recorded as the PFS. Tumor progression was evaluated by Nuclear Magnetic Resonance Imaging (MRI) every 4 weeks according to the modified Response Evaluation Criteria in Solid Tumors (mRECIST) criteria, defined as an increase of at least 20% in the sum of the diameters of viable target lesions, with the smallest sum of the diameters of viable target lesions recorded since the start of treatment used as a reference.

### 2.4 Measurement of Peripheral Serum Cytokine Levels

We collected the samples on the morning while the patients were in a fasting state. For biochemical testing, blood samples were collected using standard tubes without anticoagulants. For routine blood cell count testing, specimens were collected using heparin anticoagulant tubes. For cytokine detection, samples were also collected without anticoagulants, and the supernatant was obtained after centrifugation for testing. The concentrations of cytokines, IL-4, IL-6, IL-10, IFN-γ, TNF-α, and IL-17A) in the peripheral serum of patients in both groups were measured using a multiplex cytokine detection kit (Saiji Biotechnology, Nanchang, China). This kit quantifies cytokine levels by measuring flow cytometric fluorescence intensity. In accordance with the kit instructions, standard samples were first diluted in a concentration gradient of 1:2, 1:4, 1:8, 1:16, 1:32, 1:64, 1:128, 1:256, 1:512, 1:1024, and 1:2048. The standard samples were then analyzed using a flow cytometer to generate a standard curve. The test samples were subsequently mixed with capture bead solutions and fluorescence detection reagents, followed by incubation and analysis via a flow cytometer. The concentrations of the test samples were calculated on the basis of the standard curve.

### 2.5 Statistical Analysis

All statistical analyses were carried out using R software (version 4.3.2; R Foundation for Statistical Computing, Vienna, Austria). The ‘survival 3.8.3’ and ‘ggplot2 4.0.3’ packages were employed for data analysis and graphical display. Normality of the patient data was first assessed by the Shapiro–Wilk test. Variables following a normal distribution are presented as mean ± standard deviation (SD), whereas those with a non-normal distribution are summarized as median with interquartile range (IQR). For between-group comparisons, continuous variables were analyzed using either independent-samples *t*-tests (for normally distributed data) or Mann–Whitney U tests (for non-normal data). Categorical variables were compared by chi-square tests, with Fisher’s exact test applied when expected frequencies were small. Paired comparisons (e.g., changes in blood cell counts, liver function indices, systemic inflammatory markers, and serum cytokine levels before vs. after therapy) were evaluated using paired *t*-tests or Wilcoxon signed-rank tests, depending on data distribution. Survival curves were generated by the Kaplan–Meier method and plotted in R. Receiver operating characteristic (ROC) curves were constructed for survival outcomes, and patients were dichotomized into good- and poor-prognosis groups according to the median survival time. The optimal cut-off value for the risk score was determined by maximizing the Youden index from the ROC analysis. Prognostic factors were identified via univariate and multivariate Cox proportional-hazards regression. In all analyses, a two-sided *p* value < 0.05 was deemed statistically significant.

## 3 Results

### 3.1 Baseline Characteristics of Patients

Between 2018 and 2023, 77 HCC patients met the eligibility criteria and were sorted into the iNKT + TACE and TACE groups. The treatment and clinical data collection processes are shown in Fig. 1A. The baseline characteristics of the patients are shown in Table 1. The iNKT + TACE group consisted of 39 patients, with an average age of 60 ± 10.3 years, while the TACE group comprised 38 patients, with an average age of 57 ± 9.4 years. Before treatments, there were no significant differences in various indicators, such as biochemical test data (including liver function Child-pugh, alpha-fetoprotein levels, albumin levels, bile acid levels, etc.), BCLC stage, routine blood tests (including lymphocyte counts, neutrophil counts, monocyte counts and platelet counts), circulating inflammation indices (NLR, LMR, SII, and SIRI), or medical history (*p* > 0.05, Table 1), between the two groups.

**Table 1:** Baseline characteristics for the iNKT +TACE and TACE group.

	iNKT + TACE (*n* = 39)	TACE (*n* = 38)	*p*-Value
Age (years)	60.0 ± 10.3	57.0 ± 9.4	0.14
Sex/Male (%)	32 (82.1%)	33 (86.8%)	0.56
Cause of HCC			0.06
HBV	37 (94.9%)	31 (81.6%)	
HCV	2 (5%)	2 (5.3%)	
Other	0 (0%)	5 (13.1%)	
Liver cirrhosis			0.54
Yes	32 (82.1%)	29 (76%)	
No	7 (17.9%)	9 (24%)	
BCLC			0.47
A	4 (10.3%)	2 (5.3%)	
B	23 (58.9%)	20 (52.6%)	
C	12 (30.7%)	16 (42.1%)	
Lymphocyte (10^9^/L)	1.11 (0.74, 1.73)	1.22 (0.77, 1.62)	0.96
Neutrophil (10^9^/L)	2.84 (1.78, 3.51)	2.67 (1.63, 3.61)	0.57
Monocyte (10^9^/L)	0.33 (0.21, 0.41)	0.34 (0.26, 0.46)	0.26
Platelet (10^9^/L)	108 (75.00, 175.00)	111.50 (76.00, 188.50)	0.29
AFP (ng/mL)			0.85
>400	11 (28.3%)	10 (26.3%)	
≤400	28 (71.7%)	28 (73.7%)	
Child-Pugh			0.86
A	27 (69.2%)	27 (71%)	
B	12 (30.8%)	11 (29%)	
ALT (U/L)	26.50 (16.00, 39.30)	30.50 (22.00, 50.30)	0.25
AST (U/L)	37.00 (23.75, 48.00)	32.00 (21.75, 48.00)	0.24
Albumin (g/L)	37.20 (32.30, 40.20)	36.00 (31.40, 38.80)	0.29
Bile acid (μmol/L)	15.70 (10.10, 31.50)	26.50 (8.98, 73.60)	0.29
TBIL (μmol/L)	21.2 (11.2, 28.6)	19.35 (15.25, 28.88)	0.85
PTA (%)	78.82 ± 13.28	80.42 ± 15.33	0.25
NLR	2.14 (1.50, 3.30)	2.16 (1.70, 3.40)	0.96
LMR	3.30 (2.24, 4.05)	4.10 (2.65, 4.57)	0.060
SII	268.4 (138.67, 399.50)	224.40 (132.20, 472.10)	0.71
SIRI	0.64 (0.40, 1.08)	0.79 (0.43, 1.23)	0.71
Hypertension	6 (15.4%)	7 (18.4%)	0.72
Diabetes	8 (20.5%)	5 (13%)	0.39
Coronary heart disease	2 (5%)	1 (2.6%)	0.51

Note: HCC: hepatocellular carcinoma; HBV: hepatitis B virus; HCV: hepatitis C virus; BCLC: Barcelona Clinic Liver Cancer; AFP: alpha-fetoprotein; ALT: alanine aminotransferase; AST: aspartate aminotransferase; TBIL: total bilirubin; PTA: prothrombin activity; NLR: neutrophil-to-lymphocyte ratio; LMR: lymphocyte-to-monocyte ratio; SII: systemic immune-inflammation index; SIRI: systemic inflammation response index.

### 3.2 Comparison of PFS between the iNKT + TACE and TACE Groups

By the end of the follow-up period, 28 patients in the iNKT + TACE group had experienced tumor progression, while all 38 patients in the TACE group had tumor progression. We compared the PFS of patients in the iNKT +TACE group and the TACE group. The median PFS was 7.2 months (95% CI: 4.00–11.50 months) in the 39 patients receiving iNKT cell therapy combined with TACE, and the median PFS was 2.6 months (95% CI: 2.10–3.30 months) in the 38 patients receiving TACE alone. The PFS in the iNKT + TACE group was significantly longer than that in the TACE group (HR = 0.29, 95% CI: 0.17–0.49, *p* < 0.01; Fig. 1B).

### 3.3 Changes in Routine Blood Biochemistry Levels and Liver Function Indices in the iNKT + TACE and TACE Groups

**Figure 2:**
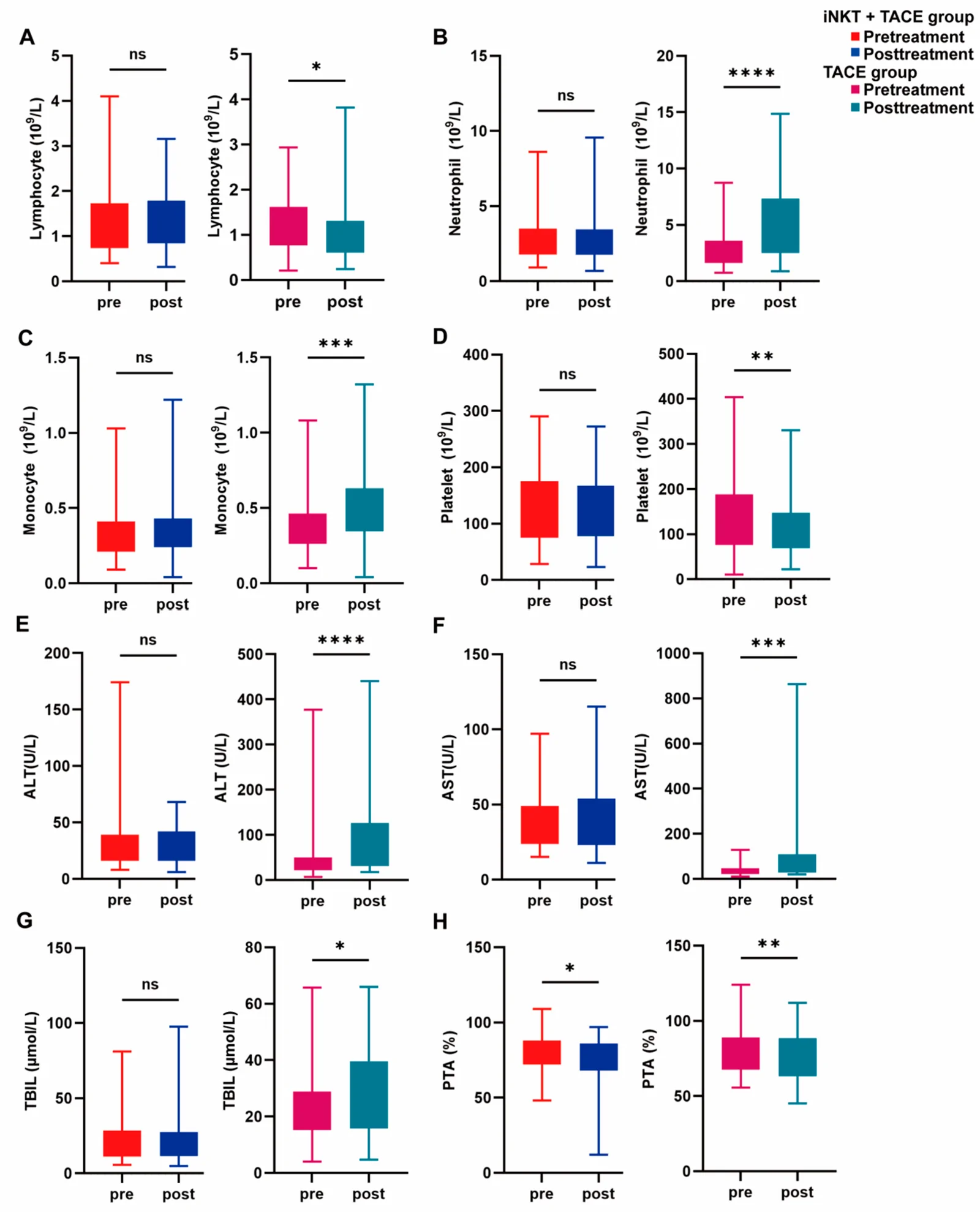
Changes in routine blood tests and liver function indices before and after treatment. (**A**) Lymphocyte counts. (**B**) Neutrophil counts. (**C**) Monocyte counts. (**D**) Platelet counts. (**E**) ALT, alanine transaminase levels. (**F**) AST, aspartate aminotransferase levels. (**G**) TBIL, total bilirubin levels. (**H**) PTA, prothrombin activity. ns, *p* > 0.05. *p < 0.05. **p < 0.01. ***p < 0.001. ****p < 0.0001.

We compared changes in peripheral blood lymphocyte, neutrophil, monocyte, and platelet counts between the two groups after treatments. The lymphocyte counts were lower in the TACE group than in the pretreatment group (0.92 × 10^9^/L vs. 1.22 × 10^9^/L, *p* = 0.032; Fig. 2A), whereas no such alteration was observed in the iNKT + TACE group. Additionally, there was no statistically significant difference in lymphocyte count between the two groups after treatment (Table 2). In the TACE group, the neutrophil count increased from 2.67 × 10^9^/L to 4.32 × 10^9^/L (*p* < 0.0001, Fig. 2B), and the monocyte count also increased (0.34 × 10^9^/L vs. 0.46 × 10^9^/L, *p* < 0.001, Fig. 2C). However, no changes were observed in either the neutrophil or monocyte count in the iNKT + TACE group. At the same time, the neutrophil and monocyte count in the TACE group were significantly higher than those in the iNKT + TACE group (Table 2). The platelet count decreased (111.5 × 10^9^/L vs. 104.5 × 10^9^/L, *p* < 0.01; Fig. 2D) in the TACE group, but no changes in the platelet count were observed in the iNKT + TACE group (Fig. 2D, Table 2).

Additionally, the changes in liver function were compared between the two groups. In the TACE group, the ALT levels increased by 15.5 U/L compared with those in the pretreatment group (*p* < 0.0001, Fig. 2E), and the AST levels increased from 32 U/L to 53 U/L (*p* < 0.001, Fig. 2F). However, no significant changes in ALT or AST levels were observed in the iNKT + TACE group. In addition, the TBIL levels also increased after treatment (19.35 μmol/L vs. 26.35 μmol/L, *p* = 0.03, Fig. 2G) in the TACE group, but they remained stable in the iNKT + TACE group. Although a decrease in prothrombin activity (PTA) was observed in both groups, the iNKT + TACE group showed only a 2% reduction in PTA compared with a 9% reduction in the TACE group (Fig. 2H). Similarly, the levels of ALT, AST, and TBIL in the iNKT + TACE group were decreased compared with those in the TACE group (Table 2).

### 3.4 Changes in Circulating Inflammatory Indices in the iNKT + TACE and TACE Groups

**Figure 3:**
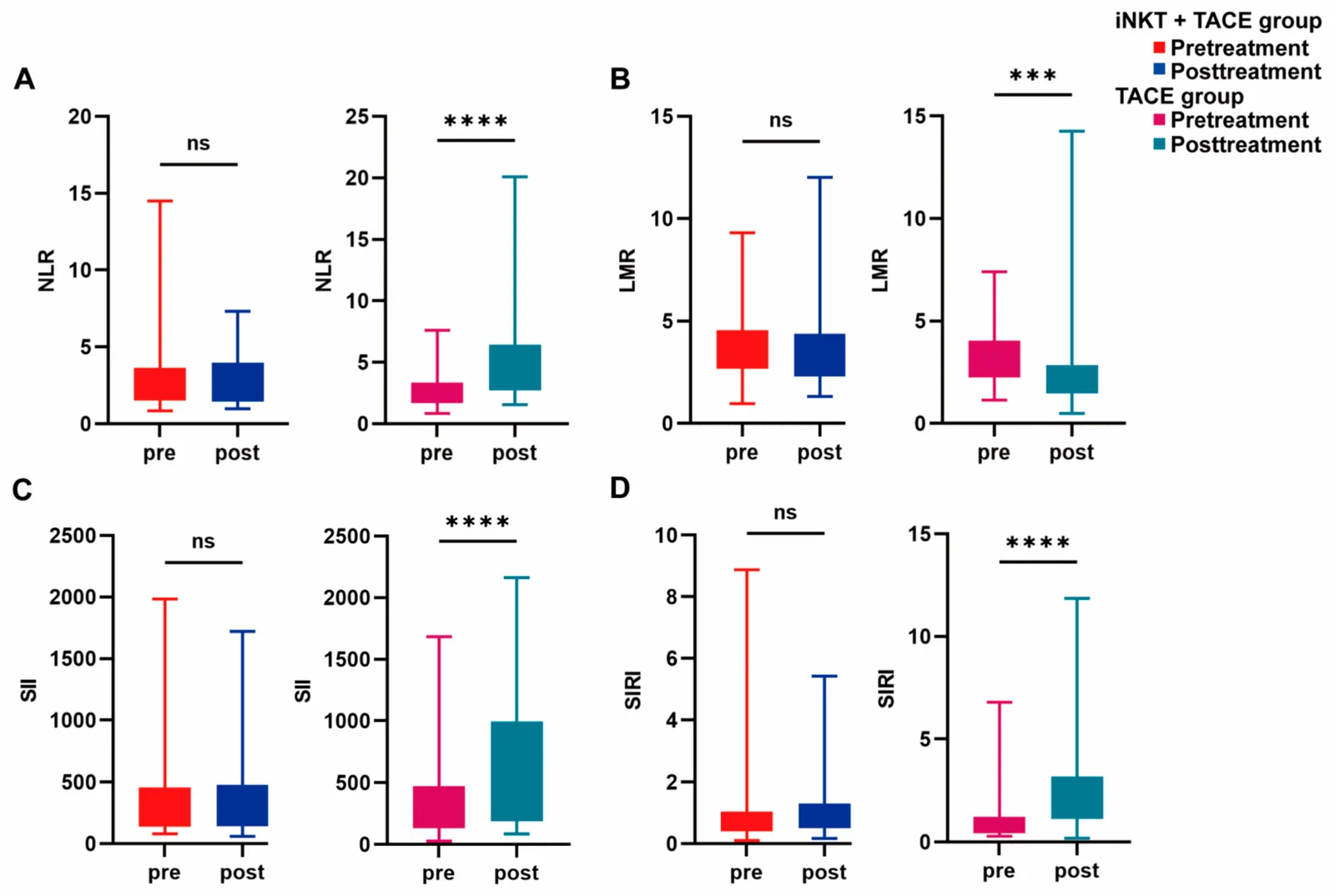
Changes in systemic inflammatory indices before and after treatment. (**A**) NLR, neutrophil-to-lymphocyte ratio. (**B**) LMR, lymphocyte-to-monocyte ratio. (**C**) SII, systemic immune-inflammation index. (**D**) SIRI, systemic inflammation response index. ns, *p* > 0.05. ***p < 0.001. ****p < 0.0001.

On the basis of the above analysis, patients in the TACE group presented increased neutrophil and monocyte counts after treatment, whereas no changes in neutrophil and monocyte counts were observed in the iNKT + TACE group. Owing to the close relationship of neutrophils and monocytes with the inflammatory response, we further analyzed the changes in circulating inflammatory indices in both groups before and after treatment. Among the 38 HCC patients in the TACE group, the NLR increased from 2.16 to 4.75 after TACE alone (*p* < 0.0001, Fig. 3A), and the SIRI increased from 0.79 to 1.95 after TACE alone (*p* < 0.0001, Fig. 3D). However, no significant change was observed in the NLR or SIRI in the iNKT + TACE group. Additionally, the SII increased by 273.64 compared with the pretreatment value (224.4 vs. 498.04, *p* < 0.0001; Fig. 3C) in the TACE group, but the SII did not significantly change in the iNKT + TACE group. In addition, the LMR decreased after TACE alone (4.1 vs. 2.04, *p* < 0.001; Fig. 3B) but remained unchanged in the iNKT + TACE group. Moreover, the levels of all four circulating inflammatory indices in the iNKT + TACE group were significantly better than those in the TACE group (Table 2).

**Table 2:** Comparison of biochemical indicators and inflammatory indices between two groups after therapy.

	iNKT + TACE (*n* = 39)	TACE (*n* = 38)	*p*-Value
Lymphocyte (10^9^/L)	1.05 (0.70, 1.44)	0.92 (0.60, 1.30)	0.47
Neutrophil (10^9^/L)	2.57 (1.77, 3.45)	4.32 (2.50, 7.32)	<0.001
Monocyte (10^9^/L)	0.32 (0.24, 0.43)	0.46 (0.35, 0.63)	0.012
Platelet (10^9^/L)	112.00 (78.00, 167.00)	104.50 (69.00, 147.25)	0.38
ALT (U/L)	32.00 (23.00, 54.00)	46.00 (28.75, 109.50)	0.015
AST (U/L)	22.00 (16.00, 42.00)	53 (31.25, 126.25)	<0.01
TBIL (μmol/L)	19.80 (11.60, 27.60)	26.35 (15.80, 39.57)	0.047
PTA (%)	76.00 (68.00, 86.00)	71.00 (63.20, 88.50)	0.94
NLR	2.20 (1.48, 3.82)	4.75 (3.74, 6.43)	<0.01
LMR	3.69 (2.42, 5.02)	2.04 (1.42, 2.54)	<0.01
SII	291 (149.55,477.78)	498.04 (188.65, 958.30)	0.019
SIRI	0.89 ± 0.1	1.95 (1.38, 3.33)	<0.01

Note: ALT: alanine aminotransferase; AST: aspartate aminotransferase; TBIL: total bilirubin; PTA: prothrombin activity; NLR: neutrophil-to-lymphocyte ratio; LMR: lymphocyte-to-monocyte ratio; SII: systemic immune-inflammation index; SIRI: systemic inflammation response index.

### 3.5 Changes in Circulating Cytokine Levels in the iNKT + TACE and TACE Groups

**Figure 4:**
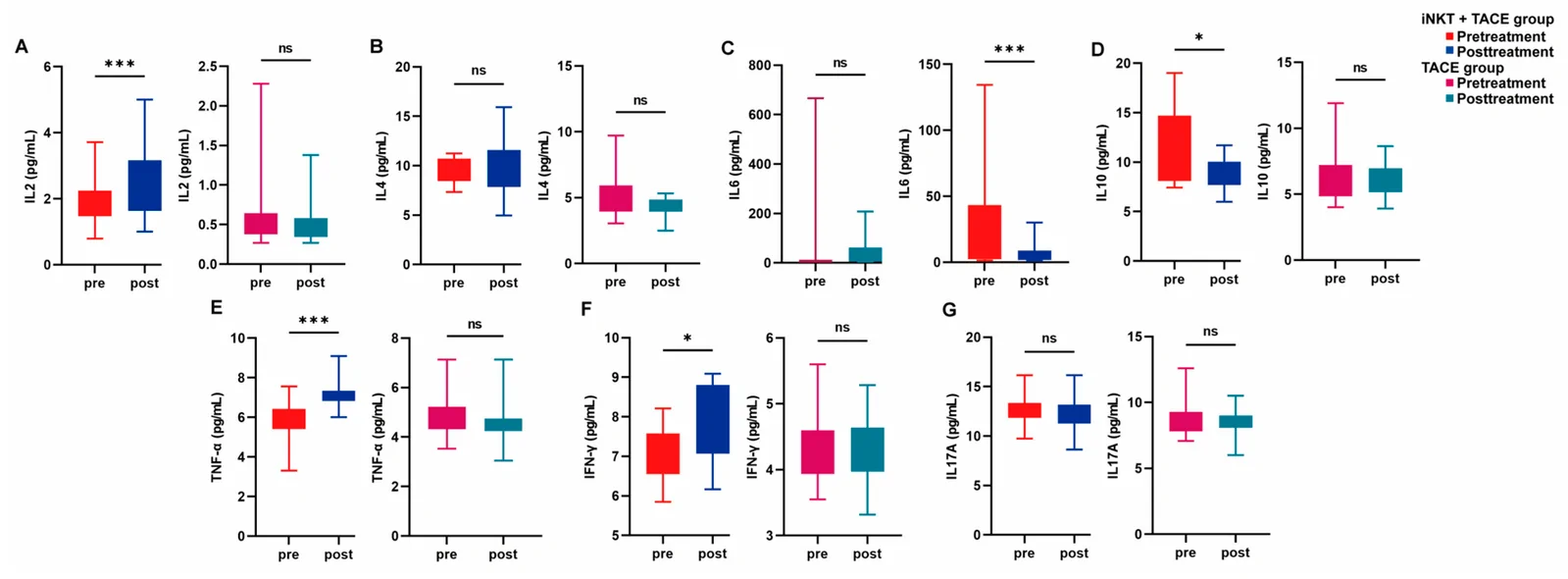
Changes in circulating cytokine levels before and after treatment. (**A**) IL-2 levels. (**B**) IL-4 levels. (**C**) IL-6 levels. (**D**) IL-10 levels. (**E**) TNF-α levels. (**F**) IFN-γ levels. (**G**) IL-17A levels. ns, *p* > 0.05. *p < 0.05. ***p < 0.001.

Because patients in the TACE group presented elevated circulating inflammatory indices after treatment and no such changes were observed in the iNKT + TACE group, we further analyzed the alterations in circulating cytokine levels in each group. After combined iNKT cell therapy and TACE, the serum IL-2 levels in HCC patients significantly increased compared with that before therapy (*p* < 0.001, Fig. 4A), whereas no significant change in posttreatment IL-2 levels was observed in the TACE group (*p* > 0.05). Compared with those in the pretreatment group, TNF-α and IFN-γ levels increased after combined treatment with iNKT cells and TACE (*p* < 0.001 and *p* < 0.05, Fig. 4E,F), whereas no significant change was observed in the serum TNF-α or IFN-γ levels after treatment with TACE alone. Interestingly, the concentrations of IL-6 and IL-10 decreased after combined treatment with iNKT cells and TACE (*p* < 0.001 and *p* < 0.05, Fig. 4C,D), but no change was observed in the TACE group. Moreover, the serum levels of IL-4 and IL-17A did not significantly change in either group (Fig. 4B,G).

Additionally, we also compared the changes in various cytokines before and after treatment between the two groups. The results are shown in Table 3. After different treatments, the changing trends of IL-2, IL-4, TNF-α, and IFN-γ in the iNKT + TACE group were higher than those in the TACE group, whereas the changing trends of IL-6, IL-10 and IL-17A were lower than those in the TACE group.

**Table 3:** Comparison of fold changes in cytokines between two groups after therapy.

	iNKT + TACE (*n* = 39)	TACE (*n* = 38)	*p*-Value
IL-2	1.35 (1.09, 1.69)	0.87 (0.36, 1.5)	0.004
IL-4	0.92 (0.7, 1.09)	0.86 (0.71, 1.07)	0.85
IL-6	0.34 (0.17, 0.63)	0.84 (0.25, 2.97)	0.004
IL-10	0.82 (0.64, 0.98)	0.97 (0.71, 1.41)	0.04
TNF-α	1.23 (1.04, 1.41)	0.98 (0.84, 1.05)	<0.001
IFN-γ	1.09 (1.03, 1.15)	0.98 (0.85, 1.08)	<0.01
IL-17A	0.94 (0.80, 1.05)	1.03 (0.93, 1.16)	0.007

Note: IL-2: Interleukin-2; IL-4: Interleukin-4; IL-6: Interleukin-6; IL-10: Interleukin-10; TNF-α: Tumor Necrosis Factor-alpha; IFN-γ: Interferon-gamma; IL-17A: Interleukin-17A.

### 3.6 Cox Regression Analysis of PFS

**Figure 5:**
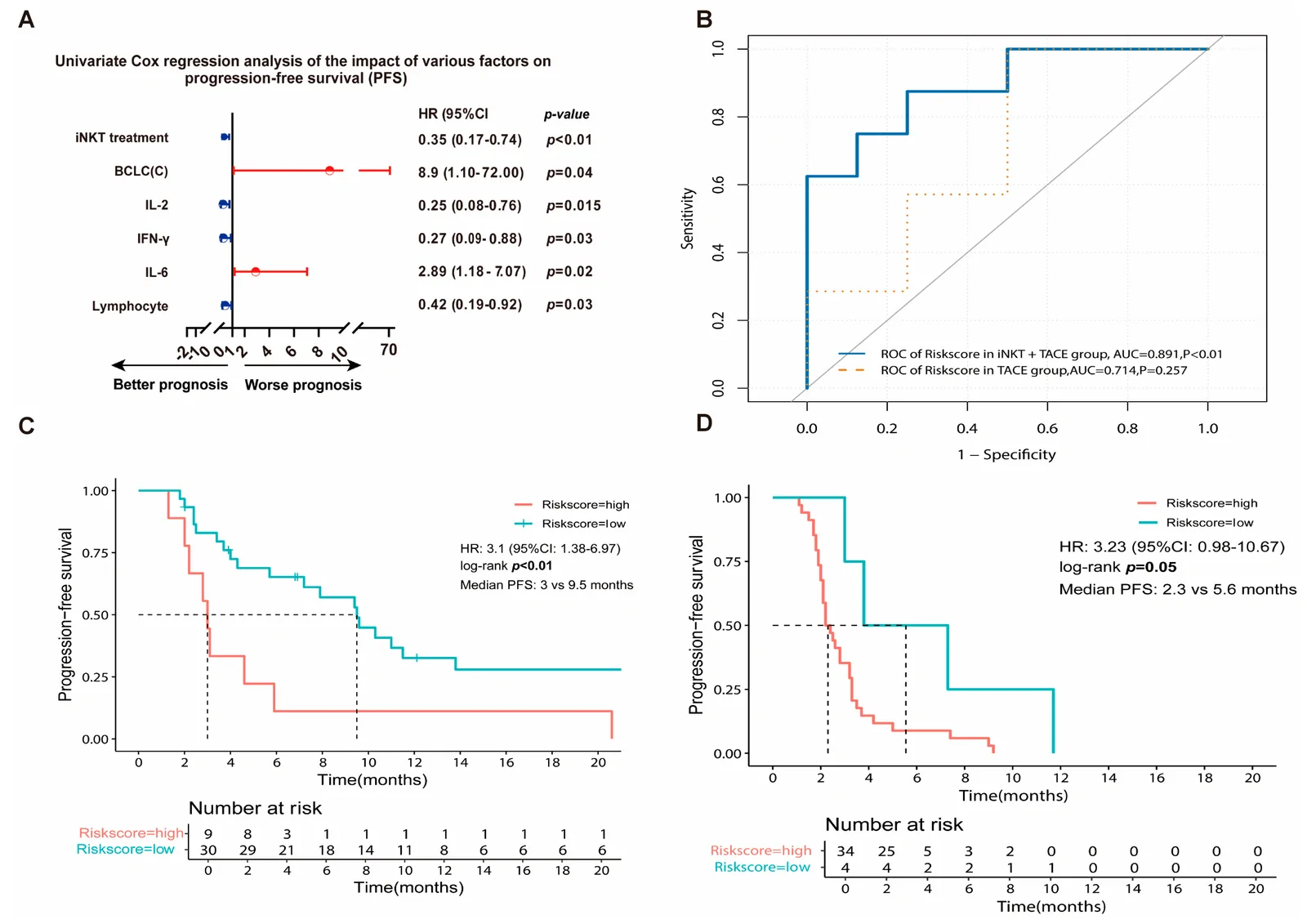
The risk score to predict patient prognoses. (**A**) Forest plot of the univariate Cox regression analysis. (**B**) ROC analysis of the risk score in both groups. (**C**,**D**) Effects of the risk score on PFS in the iNKT + TACE group (**C**) and the TACE group (**D**).

On the basis of the observed changes in circulating inflammatory indices and cytokine levels, we performed univariate Cox regression analysis to evaluate the impact of these alterations on patient PFS, which revealed that iNKT cell therapy was one of the factors influencing PFS. Compared with HCC patients who did not receive iNKT cell therapy, those who received iNKT cell therapy had a 0.65-fold reduction in the risk of tumor progression (HR = 0.35, 95% CI: 0.17–0.74, *p* < 0.01; Fig. 5A). For HCC patients with higher lymphocyte counts after treatment, the risk of tumor progression was 0.42 times that of patients with lower lymphocyte counts (HR = 0.42, 95% CI: 0.19–0.92, *p* = 0.03; Fig. 5A). In addition, univariate Cox regression revealed that higher serum levels of cytokines such as IL-2 (HR = 0.25, 95% CI: 0.08–0.76, *p* = 0.015; Fig. 5A, Supplementary Table S1) and IFN-γ (HR = 0.27, 95% CI: 0.09–0.88, *p* = 0.03; Fig. 5A, Supplementary Table S1) were associated with a reduced risk of tumor progression. In contrast, HCC patients with higher IL-6 levels had a 2.89-fold greater risk of tumor progression than those with lower IL-6 levels (HR = 2.89, 95% CI: 1.18–7.07, *p* = 0.02; Fig. 5A, Supplementary Table S1). Furthermore, BCLC stage was also a significant factor influencing PFS. Patients with a more advanced BCLC stage had a greater risk of tumor progression after treatment (HR = 8.9, 95% CI: 1.10–72.00, *p* = 0.04; Fig. 5A, Supplementary Table S1).

We next conducted multivariate Cox regression analysis incorporating the significant factors identified in the univariate Cox analysis. Higher lymphocyte counts were an independent protective factor for PFS (HR = 0.18, 95% CI: 0.04–0.76, *p* = 0.02; Table 4). In addition, higher serum IFN-γ levels were associated with a decreased risk of tumor progression (HR = 0.14, 95% CI: 0.02–0.98, *p* = 0.04; Table 4). IL-6 was identified as an independent risk factor for PFS (HR = 2.57, 95% CI: 1.03–6.86, *p* = 0.04; Table 4).

**Table 4:** Multivariate cox regression analysis.

	Multivariate Cox Regression	
	Hazard Ratio (95%CI)	*p*-Value
Lymphocyte	0.18 (0.04–0.76)	0.02
IL-2	0.32 (0.09–1.76)	0.08
IL-6	2.57 (1.03–6.86)	0.040
IFN-γ	0.14 (0.02–0.98)	0.040
BCLC (C vs. A/B)	2.50 (0.87–7.36)	0.08
iNKT treatment (Yes vs. No)	1.60 (0.36–7.68)	0.51

Note: IL-2: Interleukin-2; IL-6: Interleukin-6; IFN-γ: Interferon-gamma; BCLC: Barcelona Clinic Liver Cancer; iNKT: Invariant Natural Killer T.

### 3.7 Prognostic Prediction Based on Changes in Lymphocyte Counts, IL-6 Levels, and IFN-γ Levels in the iNKT + TACE Group

Multivariate Cox regression analysis revealed that lymphocyte counts, IL-6 levels, and IFN levels were independently associated with patient PFS. Therefore, we constructed the following risk score based on their regression coefficients: risk score = −1.7 × lymphocyte count + 0.95 × IL-6 level − 1.98 × IFN-γ level. Each variable in the formula represents the ratio of post-treatment to pre-treatment levels (post-treatment/pre-treatment). The predictive value of individual indicators, such as lymphocyte counts, IL-6 levels, and IFN-γ levels, as well as the risk score, for PFS were evaluated in the two groups. As shown in Fig. S1A and Fig. S2A, for the prediction of PFS according to IL-6 level, the area under the curve (AUC) was 0.812 (95% CI: 0.59–1.00, *p* = 0.036) in the iNKT + TACE group and 0.589 (95% CI: 0.23–0.95, *p* = 0.637) in the TACE group. The lymphocyte counts and IFN-γ levels did not reach statistical significance in the ROC analysis for the outcomes of HCC patients in either group. However, for the prediction of PFS according to risk score, the AUC was 0.891 (95% CI: 0.73–1.00, *p* < 0.01; Fig. 5B) in the iNKT + TACE group and 0.714 in the TACE group (95% CI: 0.37–1.00, *p* = 0.257; Fig. 5B), indicating that the risk score had high predictive value for the outcomes of HCC patients only in the iNKT + TACE group. Meanwhile, the prognostic predictive ability of the risk score in the iNKT group was significantly higher than that of any single indicator (Supplementary Table S2). Using a cutoff of −2.2, the sensitivity for patient stratification into good and poor prognoses in the iNKT + TACE group was 75%, and the specificity was 87.5%.

### 3.8 Impact of Changes in Lymphocyte Counts, IL-6 Levels, and IFN-γ Levels on PFS in the iNKT + TACE Group

We subsequently separately evaluated the impact of lymphocyte counts, IL-6 levels, and IFN-γ levels on patient PFS. For the prediction of PFS according to lymphocyte counts, IL-6, and IFN-γ levels, the AUC was 0.766, 0.812 and 0.766 (Supplementary Fig. S1A) in the iNKT + TACE group and only IL-6 levels could predict PFS (*p* < 0.05, Supplementary Fig. S1A). However, in the TACE group, none of lymphocyte counts, IL-6, or IFN-γ levels demonstrated predictive value for PFS (*p* > 0.05, Supplementary Fig. S2A). Patients with higher lymphocyte counts (>1.25) had a lower risk of tumor progression than those with lower lymphocyte counts (<1.25) (HR = 0.38, 95% CI: 0.15–0.96, *p* = 0.039; Supplementary Fig. S1B) in the iNKT + TACE group, but there was no association between lymphocyte counts and PFS in the TACE group (Supplementary Fig. S2B). Patients with higher IFN-γ levels (>0.96) were associated with longer PFS in the iNKT + TACE group (median PFS: 20.6 vs. 5.7 months, *p* = 0.019; Supplementary Fig. S1C), but there was no association of IFN-γ levels with PFS in the TACE group (Supplementary Fig. S2C). In addition, patients with lower levels of IL-6 (<6.79) had longer PFS in the iNKT + TACE group (median PFS: 9.6 vs. 2.9 months, *p* < 0.01; Supplementary Fig. S1D), but this correlation was not observed in the TACE group (Supplementary Fig. S2D). By combining these three indicators to calculate the risk score, it was similarly observed that in the iNKT + TACE group, HCC patients with higher risk scores had a 3.1-fold greater risk of tumor progression than those with lower risk scores (HR = 3.1, 95% CI: 1.38–6.97; *p* < 0.01; Fig. 5C). However, this correlation did not reach statistical significance in the TACE group (HR = 3.23, 95% CI: 0.98–10.67; *p* = 0.05; Fig. 5D).

## 4 Discussion

In this study, we compared changes in routine blood parameters, circulating inflammatory indices, and cytokine levels in HCC patients before and after treatment with iNKT + TACE or TACE alone. Combined treatment using iNKT cells and TACE effectively prevented the TACE-induced decrease in peripheral lymphocyte counts and increase in neutrophil and monocyte levels in HCC patients. Moreover, compared with patients in the TACE group, patients in the iNKT + TACE group presented no increase in posttreatment circulating inflammatory indices, and they had reduced levels of pro-inflammatory cytokines and elevated levels of other cytokines, such as IFN-γ and IFN-γ. Multivariate Cox analysis revealed that peripheral lymphocyte count and IL-6 and IFN-γ levels were independent influencing factors for PFS in HCC patients. iNKT cell therapy may prolong PFS by increasing the peripheral lymphocyte count, reducing the IL-6 level, and increasing the IFN-γ level. The risk score constructed on the basis of these findings predicted the prognoses of patients in the iNKT cell and TACE combination therapy group. These findings suggested that iNKT cells substantially improve TACE therapy outcomes through their dual mechanism of inflammatory suppression and antitumor immune regulation.

TACE therapy induces a localized hypoxic microenvironment in the liver, triggering the secretion of multiple proinflammatory cytokines that exacerbate regional inflammatory responses [12]. Persistent inflammatory responses promote tumor progression and are associated with poorer clinical outcomes [13]. During an inflammatory response, neutrophils and monocytes are recruited to the injury site, where they release inflammatory mediators that exacerbate the inflammatory cascade while simultaneously suppressing antitumor immune cell activity [14]. In the present study, HCC patients receiving TACE monotherapy presented significant posttreatment increases in peripheral neutrophil and monocyte counts compared with baseline levels, which may be attributed to TACE-induced localized hepatic inflammatory responses. Notably, patients in the iNKT + TACE group showed no significant elevation in neutrophil or monocyte counts posttreatment. This observation suggests that treatment with iNKT cells may improve TACE-induced local hepatic inflammatory responses. Using CCL4-induced acute hepatitis and cholestatic liver injury models, Lisbonne et al. [8] reported that iNKT cell-deficient mice present significantly greater hepatic neutrophil infiltration than control mice. These findings indicate that iNKT cells may attenuate liver inflammatory damage by suppressing neutrophil recruitment and accumulation [8,15]. Collectively, the present findings suggest that iNKT cells may ameliorate TACE-induced local hepatic inflammation by reversing the characteristic elevation of neutrophil and monocyte numbers observed following TACE monotherapy.

To further elucidate the immunomodulatory effects of iNKT cells on systemic inflammation, we quantitatively assessed three established circulating inflammatory indices, namely, the NLR, SIRI, and SII, before and after iNKT cell therapy. Previous studies have demonstrated that HCC patients exhibit elevated peripheral NLRs following TACE therapy [16]. In the present study, patients receiving TACE monotherapy exhibited significant posttreatment elevation in systemic inflammatory indices, while those treated with the iNKT cell therapy and TACE combination maintained stable levels, suggesting that iNKT cell therapy may improve the TACE-induced inflammatory response.

Our previous studies revealed that the levels of cytokines secreted by iNKT cells change after expansion *in vitro*, including increases in IFN-γ, GZMB and perforin levels and decreases in IL-4 levels, suggesting that iNKT cells may be related to the inflammatory response of the body [11]. The occurrence of the inflammatory response is closely related to changes in the levels of various proinflammatory cytokines. Therefore, we further analyzed the changes in the serum cytokine levels of patients before and after iNKT cell treatment. Combining iNKT cell therapy with TACE treatment reduced the serum levels of IL-6 and IL-10. IL-6, a pleiotropic proinflammatory cytokine, is a critical mediator across multiple stages of HCC pathogenesis by orchestrating tumor cell proliferation, driving the transformation of hepatocytes into cancer progenitor cells, and promoting metastatic dissemination [17]. IL-10, another key immunoregulatory cytokine, suppresses antitumor immune responses and actively contributes to tumor invasion and metastatic processes. In addition to reducing IL-6 and IL-10 levels, iNKT cell therapy also increased the serum levels of IFN-γ and TNF-α, which are cytokines secreted by T-cells, NK cells and other immune cells with strong killing effects on tumor cells. Collectively, these findings indicate that iNKT cells exert dual therapeutic benefits by simultaneously mitigating TACE-induced inflammatory responses through the downregulation of proinflammatory cytokine production while enhancing antitumor immunity via the secretion of cytotoxic effector molecules, thereby addressing both the inflammatory and neoplastic components of HCC progression.

On the basis of the changes in cytokine levels, multivariate Cox regression revealed that decreases in IL-6 levels, increases in IFN-γ levels, and increases in lymphocyte counts were correlated with the prognoses of HCC patients. Moreover, the prognostic risk score calculated from these three biomarkers (IL-6 level reduction, IFN-γ level elevation, and lymphocyte number increase) effectively predicted the clinical outcomes of HCC patients in the iNKT + TACE group. In summary, lymphocyte count, IL-6 level, and IFN-γ are relatively easy to obtain. These indicators may be more suitable for screening patients for iNKT + TACE therapy and for monitoring subsequent treatment efficacy. We have also emphasized that these findings are preliminary and require further prospective validation. A decrease in lymphocyte count typically reflects impaired immune responsiveness and diminished antitumor efficacy [18], which has been consistently associated with poorer clinical outcomes across patients with various malignancies [19]. Similarly, the present study demonstrated that patients with lower posttreatment lymphocyte counts present increased risks of tumor progression. Moreover, iNKT cell therapy combined with TACE effectively attenuated TACE-induced lymphocytopenia and was associated with prolonged PFS. Previous studies have shown that iNKT cells exhibit broad immunomodulatory capacities, demonstrating dual functionality in both activating effector immune cells (including NK cells, CD8^+^ T-cells, and B lymphocytes) and suppressing immunosuppressive populations (such as myeloid-derived suppressor cells [MDSCs] and tumor-associated macrophages [TAMs]) [20,21]. On the basis of these findings, we propose that adoptively transferred iNKT cells may exert dual therapeutic effects by simultaneously ameliorating TACE-induced inflammatory responses and modulating systemic immune function to enhance antitumor immunity.

Although the present study demonstrated that iNKT cell therapy combined with TACE ameliorated TACE-induced inflammatory responses, several limitations should be acknowledged. First, the present study was limited in that it compared peripheral lymphocyte count changes between treatment groups without further characterization of lymphocyte subset distributions or functional alterations. Second, the relatively small sample size of the present HCC cohort may limit the statistical power of the findings, necessitating future validation in larger patient populations. Finally, the present study did not investigate the underlying mechanisms of iNKT cells in modulating inflammation and immune responses, warranting further exploration in the future.

## 5 Conclusions

In conclusion, the present study confirmed that iNKT cell therapy combined with TACE reverses the increase in neutrophil and monocyte counts, reduces the serum levels of IL-6 and IL-10, and improves the inflammatory response caused by TACE. Additionally, iNKT cells prevent TACE-induced lymphocytopenia while augmenting IFN-γ and TNF-α production, thereby enhancing antitumor immunity and reinforcing therapeutic synergy. This synergistic strategy transforms TACE from a purely palliative intervention into a comprehensive therapeutic approach that addresses its current limitations and enhances patient benefit. Critically, the combined evaluation of elevated lymphocyte counts, decreased IL-6 levels, and increased IFN-γ concentrations constitutes a potent predictive signature for clinical outcomes in HCC patients receiving iNKT cell therapy combined with TACE.

## Data Availability

All data is available within the Article, Supplementary Information or available from the authors upon reasonable request.

## Supplementary Materials

The supplementary material is available online at https://www.techscience.com/doi/10.32604/or.2026.082815/s1.

## Abbreviations

AFP	alpha-fetoprotein
ALT	alanine transaminase
BCLC	Barcelona Clinic Liver Cancer
DCR	disease control rate
FasL	Fas ligand
HCC	hepatocellular carcinoma
IL	Interleukin
IFN-γ	Gamma interferon
iNKT	invariant natural killer T
KM	Kaplan-Meier
LMR	lymphocyte-to-monocyte ratio
MDSCs	myeloid-derived suppressor cells
NLR	neutrophil-to-lymphocyte ratio
ORR	objective response rate
OS	overall survival
PFS	progression-free survival
PTA	prothrombin activity
QoL	quality of life
SII	systemic immune-inflammation index
SIRI	systemic inflammation response index
TACE	Transarterial chemoembolization
TAMs	tumor-associated macrophages
TCR	T-cell receptor
TRAIL	TNF-related apoptosis-inducing ligand
