# Supplementary material for: Invariant Natural Killer T Cell Therapy Attenuates Systemic Inflammation and Improves Transarterial Chemoembolization Outcomes in Hepatocellular Carcinoma
Source: Oncol Res. 2026 Aug 13;34(9):26. doi: 10.32604/or.2026.082815 (PMC13494492; doi:10.32604/or.2026.082815)
Supplement: Supplementary file 1 [file OncolRes-34-82815-s001.zip › TSP_OR_82815-s001.docx]

**Supplementary Figure S1:** The ability of the lymphocyte counts, IFN-γ levels and IL-6 levels to predict patient prognoses in iNKT+TACE group. A. ROC analysis of the lymphocyte counts, IFN-γ levels and IL-6 levels in the iNKT+TACE group. B-D. Effects of the lymphocyte counts (B), IFN-γ levels (C) and IL-6 levels (D) on PFS in the iNKT + TACE group.

**Supplementary Figure S2:** The ability of the lymphocyte counts, IFN-γ levels and IL-6 levels to predict patient prognoses in TACE group. A. ROC analysis of the lymphocyte counts, IFN-γ levels and IL-6 levels in the TACE group. B-D. Effects of the lymphocyte counts (B), IFN-γ levels (C) and IL-6 levels (D) on PFS in the TACE group.

**Supplementary Table S1:** Univariate cox regression analysis.

|  | **Univariate cox regression** | |
| --- | --- | --- |
|  | **HR (95%CI)** | ***p*-value** |
| NLR | 2.5 (0.59, 10.65) | 0.21 |
| LMR | 0.62 (0.24, 1.61) | 0.32 |
| SII | 1.54 (0.77, 3.07) | 0.23 |
| SIRI | 1.47 (0.73, 2.98) | 0.28 |
| **Lymphocyte** | **0.42 (0.19, 0.92)** | **0.03** |
| Neutrophil | 1.98 (0.86, 4.58) | 0.11 |
| Monocyte | 1.78 (0.89, 3.55) | 0.1 |
| Platelet | 0.46 (0.21, 1.02) | 0.056 |
| **IL-2** | **0.25(0.079, 0.76)** | **0.015** |
| IL-4 | 1.15(0.45, 2.94) | 0.77 |
| **IL-6** | **2.89 (1.18, 7.07)** | **0.02** |
| IL-10 | 2.83 (0.65,12.36) | 0.17 |
| TNF-α | 0.47(0.21, 1.1) | 0.06 |
| **IFN-γ** | **0.27 (0.09, 0.88)** | **0.03** |
| IL17A | 0.37 (0.13, 1.02) | 0.054 |
| MVI | 1.62(0.86,4.76) | 0.31 |
| **BCLC C** | **8.9 (1.1,72)** | **0.04** |
| BCLC B | 3.5 (0.47, 26.3) | 0.22 |
| Age | 0.53 (0.23, 1.21) | 0.13 |
| Sex | 1.25 (0.38,4.1) | 0.72 |
| Child-Pugh | 1.48(0.29,7.4) | 0.63 |
| **iNKT treatment Yes vs No** | **0.35 (0.17, 0.74)** | **<0.01** |

Note: NLR: neutrophil-to-lymphocyte ratio; LMR: lymphocyte-to-monocyte ratio; SII: systemic immune-inflammation index; SIRI: systemic inflammation response index; IL-2: Interleukin-2; IL-4: Interleukin-4; IL-6: Interleukin-6; IL-10: Interleukin-10; TNF-α: Tumor Necrosis Factor-alpha; IFN-γ: Interferon-gamma; IL-17A: Interleukin-17A. MVI: microvascular invasion; BCLC: Barcelona Clinic Liver Cancer.

**Supplementary Table S2: C-index of individual indicators and the Riskscore**

|  | **C-index** | |
| --- | --- | --- |
|  | **iNKT+TACE group** | **TACE group** |
| Lymphocyte | 0.52 | 0.53 |
| IL-6 | 0.63 | 0.52 |
| IFN-γ | 0.62 | 0.56 |
| Riskscore | 0.69 | 0.6 |

Note: IL-6: Interleukin-6; IFN-γ: Interferon-gamma.
